# Early Cumulative Fluid Balance and Outcomes in Pediatric Allogeneic Hematopoietic Cell Transplant Recipients With Acute Respiratory Failure: A Multicenter Study

**DOI:** 10.3389/fonc.2021.705602

**Published:** 2021-07-20

**Authors:** Colin J. Sallee, Lincoln S. Smith, Courtney M. Rowan, Susan R. Heckbert, Joseph R. Angelo, Megan C. Daniel, Shira J. Gertz, Deyin D. Hsing, Kris M. Mahadeo, Jennifer A. McArthur, Julie C. Fitzgerald

**Affiliations:** ^1^ Division of Pediatric Critical Care Medicine, Department of Pediatrics, Seattle Children’s Hospital, University of Washington, Seattle, WA, United States; ^2^ Division of Critical Care, Department of Pediatrics, Riley Hospital for Children, Indiana University School of Medicine, Indianapolis, IN, United States; ^3^ Department of Epidemiology, University of Washington School of Public Health, Seattle, WA, United States; ^4^ Renal Section, Department of Pediatrics, Texas Children’s Hospital, Baylor College of Medicine, Houston, TX, United States; ^5^ Division of Critical Care, Department of Pediatrics, Nationwide Children’s Hospital, The Ohio State University, Columbus, OH, United States; ^6^ Division of Pediatric Critical Care, Department of Pediatrics, Saint Barnabas Medical Center, Livingston, NJ, United States; ^7^ Division of Critical Care, Department of Pediatrics, Weil Cornell Medical College, New York Presbyterian Hospital, New York City, NY, United States; ^8^ Stem Cell Transplantation and Cellular Therapy, Children’s Cancer Hospital, University of Texas at MD Anderson Cancer Center, Houston, TX, United States; ^9^ Division of Critical Care, Department of Pediatrics, St Jude Children’s Research Hospital, Memphis, TN, United States; ^10^ Division of Critical Care, Department of Anesthesiology and Critical Care, Children’s Hospital of Philadelphia, University of Pennsylvania Perelman School of Medicine, Philadelphia, PA, United States

**Keywords:** artificial respiration, critical illness, hematopoietic stem cell transplant, respiratory failure, water-electrolyte balance, renal replacement therapy, fluid overload

## Abstract

**Objectives:**

To evaluate the associations between early cumulative fluid balance (CFB) and outcomes among critically ill pediatric allogeneic hematopoietic cell transplant (HCT) recipients with acute respiratory failure, and determine if these associations vary by treatment with renal replacement therapy (RRT).

**Methods:**

We performed a secondary analysis of a multicenter retrospective cohort of patients (1mo - 21yrs) post-allogeneic HCT with acute respiratory failure treated with invasive mechanical ventilation (IMV) from 2009 to 2014. Fluid intake and output were measured daily for the first week of IMV (day 0 = day of intubation). The exposure, day 3 CFB (CFB from day 0 through day 3 of IMV), was calculated using the equation [Fluid in – Fluid out] (liters)/[PICU admission weight](kg)*100. We measured the association between day 3 CFB and PICU mortality with logistic regression, and the rate of extubation at 28 and 60 days with competing risk regression (PICU mortality = competing risk).

**Results:**

198 patients were included in the study. Mean % CFB for the cohort was positive on day 0 of IMV, and increased further on days 1-7 of IMV. For each 1% increase in day 3 CFB, the odds of PICU mortality were 3% higher (adjusted odds ratio (aOR) 1.03, 95% CI 1.00-1.07), and the rate of extubation was 3% lower at 28 days (adjusted subdistribution hazard ratio (aSHR) 0.97, 95% CI 0.95-0.98) and 3% lower at 60 days (aSHR 0.97, 95% CI 0.95-0.98). When day 3 CFB was dichotomized, 161 (81%) had positive and 37 (19%) had negative day 3 CFB. Positive day 3 CFB was associated with higher PICU mortality (aOR 3.42, 95% CI 1.48-7.87) and a lower rate of extubation at 28 days (aSHR 0.30, 95% CI 0.18-0.48) and 60 days (aSHR 0.30, 95% 0.19-0.48). On stratified analysis, the association between positive day 3 CFB and PICU mortality was significantly stronger in those not treated with RRT (no RRT: aOR 9.11, 95% CI 2.29-36.22; RRT: aOR 1.40, 95% CI 0.42-4.74).

**Conclusions:**

Among critically ill pediatric allogeneic HCT recipients with acute respiratory failure, positive and increasing early CFB were independently associated with adverse outcomes.

## Introduction

Pediatric patients post-allogeneic hematopoietic cell transplantation (HCT) are high utilizers of pediatric intensive care unit (PICU) resources (1), particularly invasive mechanical ventilation (IMV) (2). Acute respiratory failure (ARF) in this population represents a leading cause of admission to the PICU (3–5) and mortality among patients with ARF requiring IMV remains unacceptably high at 42-60% (6–10). Therefore, identification of modifiable factors to improve survival are paramount.

There is consistent and reproducible evidence of an independent association between positive fluid balance and adverse outcomes in critically ill adult and pediatric patients (11–13). Observational studies among critically ill pediatric patients with ARF indicate associations between positive fluid balance and oxygen deficits, fewer ventilator free days, and higher mortality (14–19). The Fluid and Catheter Treatment Trial (FACTT) in adults with ARF demonstrated that a conservative fluid management strategy improved lung function and shortened duration of IMV (20). Valentine et al. (16) showed that fluid balance patterns in a pediatric cohort with ARF were similar to the liberal fluid management arm, rather than the conservative arm of the FACTT trial. This suggested that known associations between positive fluid balance and poor outcomes in patients with ARF had not influenced pediatric practice. Notably, this analysis excluded pediatric HCT recipients.

Studies in pediatric HCT recipients indicate that fluid overload is a predominant indication for RRT (21). Similar to those with ARF, mortality estimates are dismal in pediatric HCT recipients receiving renal replacement therapy (RRT) at 54-77% (21–23). Furthermore, when patients are treated with both IMV and RRT, survival is especially poor (8, 24).

Data are sparse on the effect of fluid balance on outcomes in pediatric allogeneic HCT recipients with ARF. Furthermore, the role of RRT in managing fluid balance in HCT recipients with ARF is not well defined. Our objective was to measure the associations between early cumulative fluid balance, PICU mortality, and the rate of extubation in pediatric allogeneic HCT recipients with ARF requiring IMV. We also sought to determine whether these associations differed according to receipt of RRT. We hypothesized that both positive and increasing cumulative fluid balance early in the course of IMV would be independently associated with adverse outcomes. We also hypothesized that the relationship between early cumulative fluid balance and outcomes would differ depending on receipt of RRT.

## Materials and Methods

### Design, Setting, and Patients

The current investigation represents a secondary analysis of a multicenter observational retrospective cohort study of patients post-allogeneic HCT admitted to the PICU with the diagnosis of ARF from 2009 to 2014 (7). The parent study was coordinated by the HCT-Cancer Immunotherapy subgroup of the Pediatric Acute Lung Injury and Sepsis Investigators (PALISI) network. Institutional Review Board (IRB) approval was obtained from the 12 participating pediatric centers. Each pediatric center contributed up to 25 of their most recent consecutive HCT patients requiring IMV in the study period. Additional IRB approval was not required for the current analysis as the dataset was de-identified.

**Figure 1:**
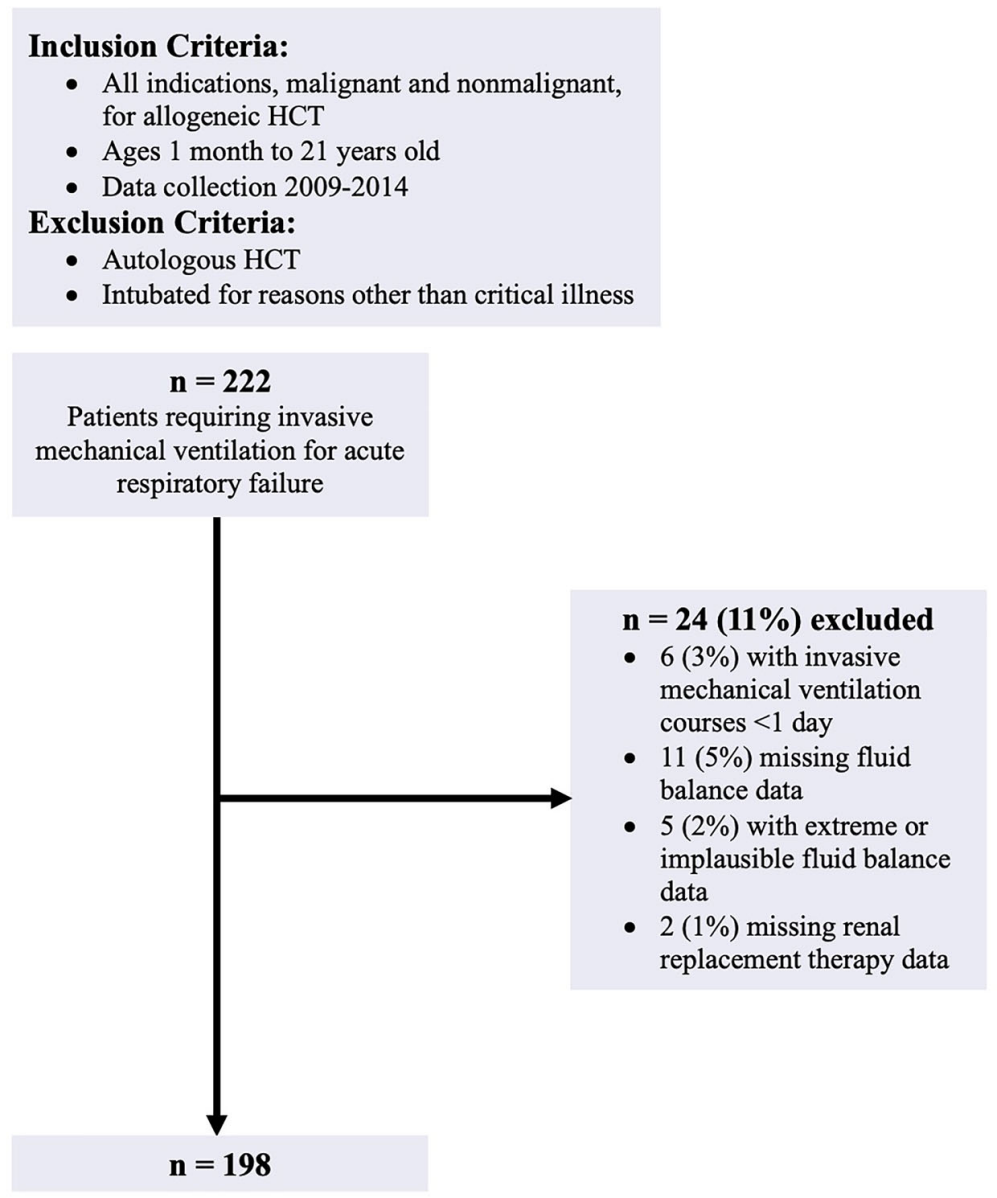
Patient flow diagram.

There were 222 allogeneic HCT recipients aged 1 month to 21 years old with ARF requiring IMV in the parent analysis. All indications, malignant and nonmalignant, for allogeneic HCT were included. Patients who received their HCT prior to January 1, 2009, had an autologous transplant, or were intubated for reasons other than critical illness (i.e. procedure) were excluded from the parent study. For the present investigation, we additionally excluded patients receiving <1 day of IMV, patients with incomplete fluid balance data, and patients missing RRT data. During dataset review, 5 additional patients were excluded due to daily fluid intake or output values that were extreme and implausible, thus were presumed to be data entry errors. With these exclusion criteria, the final cohort was 198 patients (**Figure 1**).

### Data Collection

Data recorded included demographics such as PICU admission weight, age, sex, and race. Transplant specific variables included days from HCT to PICU admission, number of transplants, diagnosis leading to transplant, source of hematopoietic progenitor cells, related or unrelated donor, and pre-transplant conditioning. Clinical variables measured included the use of supplemental oxygen 7 days prior to IMV or non-invasive positive pressure (NIPPV) prior to IMV. Treatment with inhaled nitric oxide (iNO), vasoactive or inotropic infusions, RRT, and the presence of a positive respiratory pathogen were recorded. RRT was defined as receipt of peritoneal dialysis, intermittent hemodialysis, or continuous renal replacement therapy. The timing, indication, and duration for RRT were not recorded in the parent study.

### Outcomes

The primary outcome of interest was PICU mortality, defined as all-cause death prior to discharge from the PICU. Duration of IMV was also a relevant outcome of interest, and was framed within the context of time to successful extubation. Liberation from IMV for >48 hours defined successful extubation. Therefore, the secondary outcomes were the rate of extubation within 28 days and 60 days. In the parent study, the median length of ventilation was longer than for a general PICU population and many patients were successfully extubated after 28 days. Therefore, we felt it was relevant to evaluate this outcome using a 60-day follow-up period.

### Definitions of Exposures

Daily fluid balance was measured as the total 24 hour input minus output on each study day normalized per kilogram (kg) PICU admission weight. Cumulative fluid balance (CFB) was then measured as the daily running 24 hour total (input-output) normalized per kg summed across consecutive days starting on day of intubation (day 0). The last available consecutive day of fluid balance data was the earlier of day 7 of IMV or the last day of IMV. CFB on a given day between day 0 and day 7 was then defined by percentage (%) CFB based upon the equation by Goldstein et al. (25):

$$\%CFB=\frac{\left[Fluid\;in-Fluid\;out\;(liters)\right]}{PICU\;admission\;weight\;(kg)}*100$$

The primary exposure of interest was % CFB from day 0 through day 3 of IMV (referred to hereafter as day 3 CFB). Day 3 CFB was evaluated as a continuous variable, and also categorically, either dichotomized as positive or negative, or as multiple categories (negative fluid balance, ≥0 to <10%, ≥10 to <20%, or ≥20%, respectively). There were 26 patients intubated for <3 days with CFB measured through the final day of IMV. For these patients without day 3 fluid balance data, we used the CFB over the duration of their IMV course. For the purposes of this investigation, cumulative fluid balance (CFB), fluid balance, and fluid accumulation replaced the term “fluid overload”.

### Statistical Analysis

Categorical variables were summarized with frequency (%). Continuous variables were expressed as mean ± standard deviation (SD) when normally distributed or median with interquartile range (IQR) otherwise. Patient demographic data were summarized overall and by the exposure, positive *vs.* negative day 3 CFB.

Day 3 CFB was evaluated as a continuous, dichotomous (positive *vs.* negative), grouped-linear (or ordinal), and categorical variable (with negative day 3 CFB as reference) when assessing the relationship with the relevant outcomes. The CFB categories were analyzed in a grouped-linear fashion to account for the hypothesis that each increase in CFB category would confer worse outcomes (test of linear trend).

We evaluated the association between day 3 CFB and the primary outcome, PICU mortality, with bivariate and multivariable logistic regression models. We then evaluated the association between day 3 CFB and the secondary outcomes, rate of extubation censored at 28 and 60 days, with bivariate and multivariable Fine and Gray competing risk regression (26). Competing risk regression is a form of time to event analysis with the event of interest being extubation and PICU mortality serving as the competing risk. Competing risk refers to the possibility that instead of extubation, a competing event could be observed, in this case, PICU mortality. In traditional Cox regression, patients who die would be censored and their censoring would be assumed to be non-informative, which would be inappropriate in the present study. Therefore, the final analysis produced a subdistribution hazard ratio (SHR) that estimates the hazard of day 3 CFB on the probability of extubation, while accounting for the fact that mortality would preclude the occurrence of extubation. We assessed for effect modification by treatment with RRT on the associations of interest using interaction terms in the multivariable regression models. We determined effect modification by statistical significance of the interaction term between day 3 CFB and RRT in our multivariable regression models.

**Figure 2:**
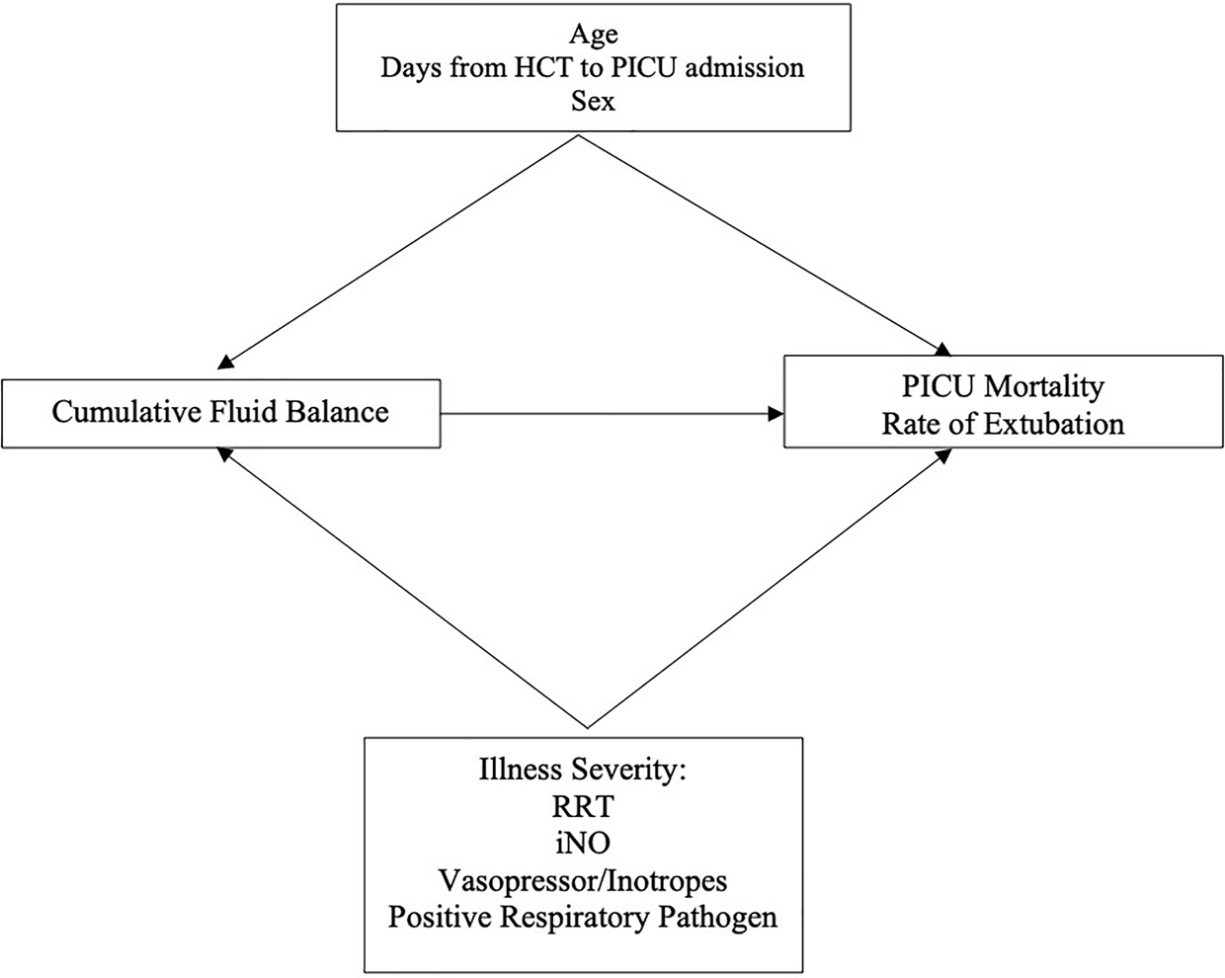
Directed Acyclic Graph. HCT, Hematopoietic Cell Transplant; RRT, Renal Replacement Therapy; iNO, Inhaled Nitric Oxide.

We adjusted for several covariates defined *a priori* (age in years, sex, days from HCT to PICU admission, presence of a positive respiratory pathogen, and treatment with iNO, vasoactive or inotropic infusions, and RRT) in the multivariable models (**Figure 2**). We conducted a *post-hoc* sensitivity analysis excluding the 26 patients with IMV courses <3 days performing the same models described above. In all multivariable models, a p-value <0.05 was considered statistically significant. All statistical analyses were performed using STATA 16.1 (College Station, TX).

## Results

Among the 198 patients in the cohort, 161 (81%) had positive CFB and 37 (19%) had negative CFB through day 3 of IMV. Defining categories of day 3 CFB, 19% were negative, 38% were ≥0 to <10%, 24% were ≥10 to <20%, and 19% were ≥20%. Baseline demographic, clinical, and transplant characteristics are illustrated in **Table 1** stratified by the exposure, positive or negative day 3 CFB. Relative to the negative day 3 CFB group, patients with positive day 3 CFB were younger (9.1 years *vs.* 13.2 years), weighed less (30.9 kg *vs.* 50.4 kg), had more time from HCT to PICU admission (49 days *vs.* 34 days), and notably, were less frequently treated with RRT (34% vs. 54%). Additionally, fewer positive day 3 CFB patients were transplanted for malignancy (50% *vs.* 68%) and conditioned with total body irradiation (32% *vs.* 49%) (**Table 1**).

**Table 1:** Demographic, hematopoietic cell transplant, and PICU characteristics of study population.

Variable	Total Cohort	Positive Day 3 CFB	Negative Day 3 CFB
	N = 198	N=161	N=37
**Demographics**			
Age, median (IQR), yr	10.2 (2.6-16.1)	9.1 (2.2-16.0)	13.2 (6.9-16.8)
Weight, median (IQR), kg	33.8 (13.5-58.6)	30.9 (13.1-55.0)	50.4 (25.0-66.0)
Female, n (%)	84 (42)	68 (42)	16 (43)
Race, n (%)			
African-American or Black	24 (12)	17 (11)	7 (19)
Asian	13 (7)	11 (7)	2 (5)
Other	57 (29)	45 (27)	12 (33)
White	104 (52)	88 (55)	16 (43)
**Transplant Characteristics**			
Transplant for malignancy, n (%)	105 (53)	80 (50)	25 (68)
Source of transplant, n (%)			
Bone marrow	89 (45)	71 (44)	18 (49)
Cord blood	77 (39)	66 (41)	11 (30)
Peripheral blood	32 (16)	24 (15)	8 (21)
Related donor, n (%)	44 (22)	34 (21)	10 (27)
Conditioning with fludarabine, n (%)	95 (48)	76 (47)	19 (51)
Conditioning with TBI, n (%)	70 (35)	52 (32)	18 (49)
Transplant number, n (%)			
First	167 (84)	136 (85)	31 (84)
Second	29 (15)	23 (14)	6 (16)
Third	2 (1)	2 (1)	0 (0)
HCT to PICU, median (IQR), days	44.5 (17-116)	49 (16-116)	34 (18-118)
**PICU Characteristics**			
Oxygen 7 days prior to IMV, n (%)	76 (38)	65 (40)	11 (30)
NIPPV prior to IMV, n (%)	99 (50)	81 (50)	18 (49)
Positive respiratory pathogen, n (%)	72 (36)	58 (35)	14 (38)
Renal replacement therapy, n (%)	75 (38)	55 (34)	20 (54)
Vasopressors/Inotropes, n (%)	148 (75)	120 (75)	28 (76)
Inhaled nitric oxide, n (%)	57 (29)	45 (28)	12 (32)

CFB, cumulative fluid balance; Age, years; Weight, PICU admission weight in kilograms; TBI, total body irradiation; HCT, hematopoietic cell transplantation; O2, supplemental oxygen; NIPPV, non-invasive positive pressure ventilation; RRT, renal replacement therapy.

**Figure 3:**
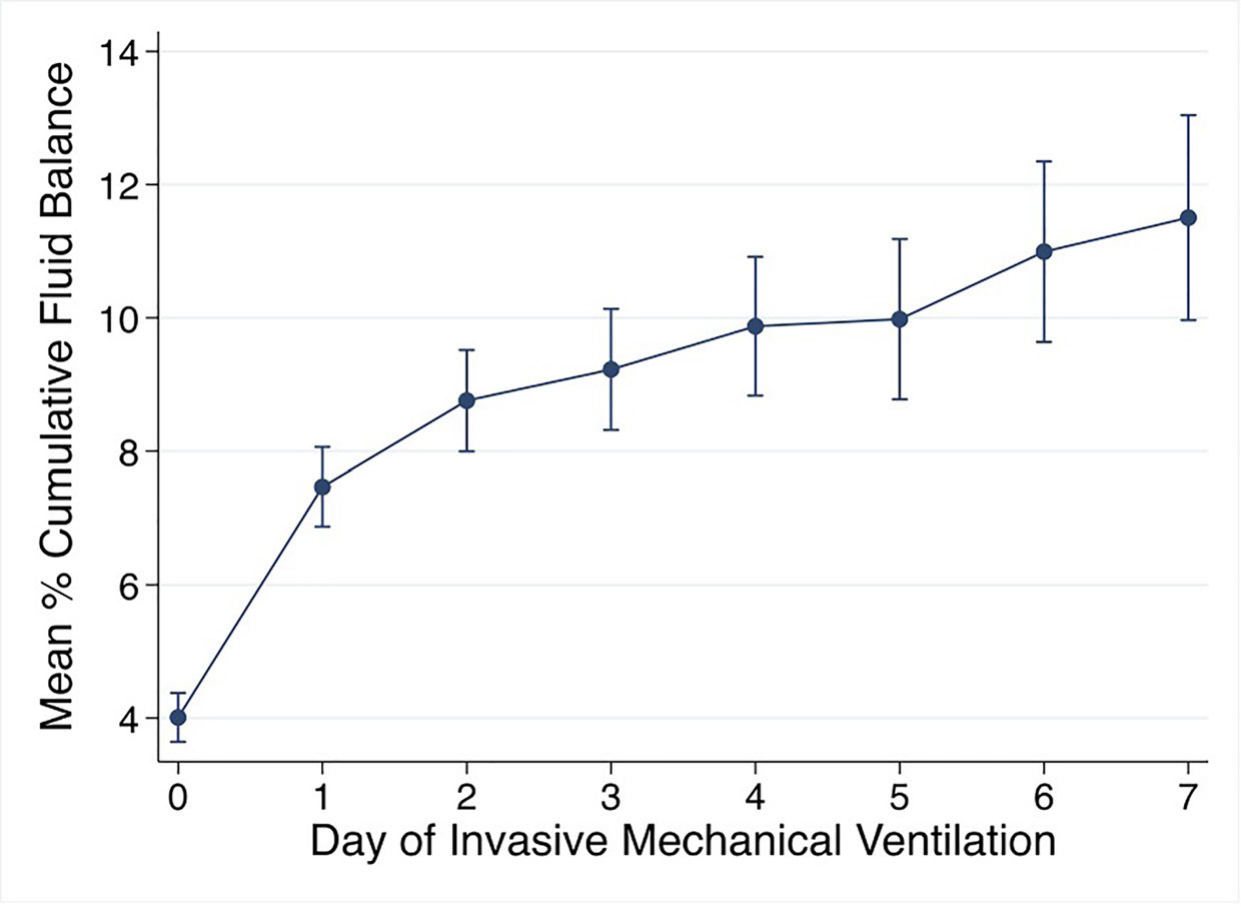
Mean % cumulative fluid balance by day of invasive mechanical ventilation. % cumulative fluid balance =[Fluid in-Fluid out (liters)]/[PICU admission weight (kg)] *100.

**Figure 4:**
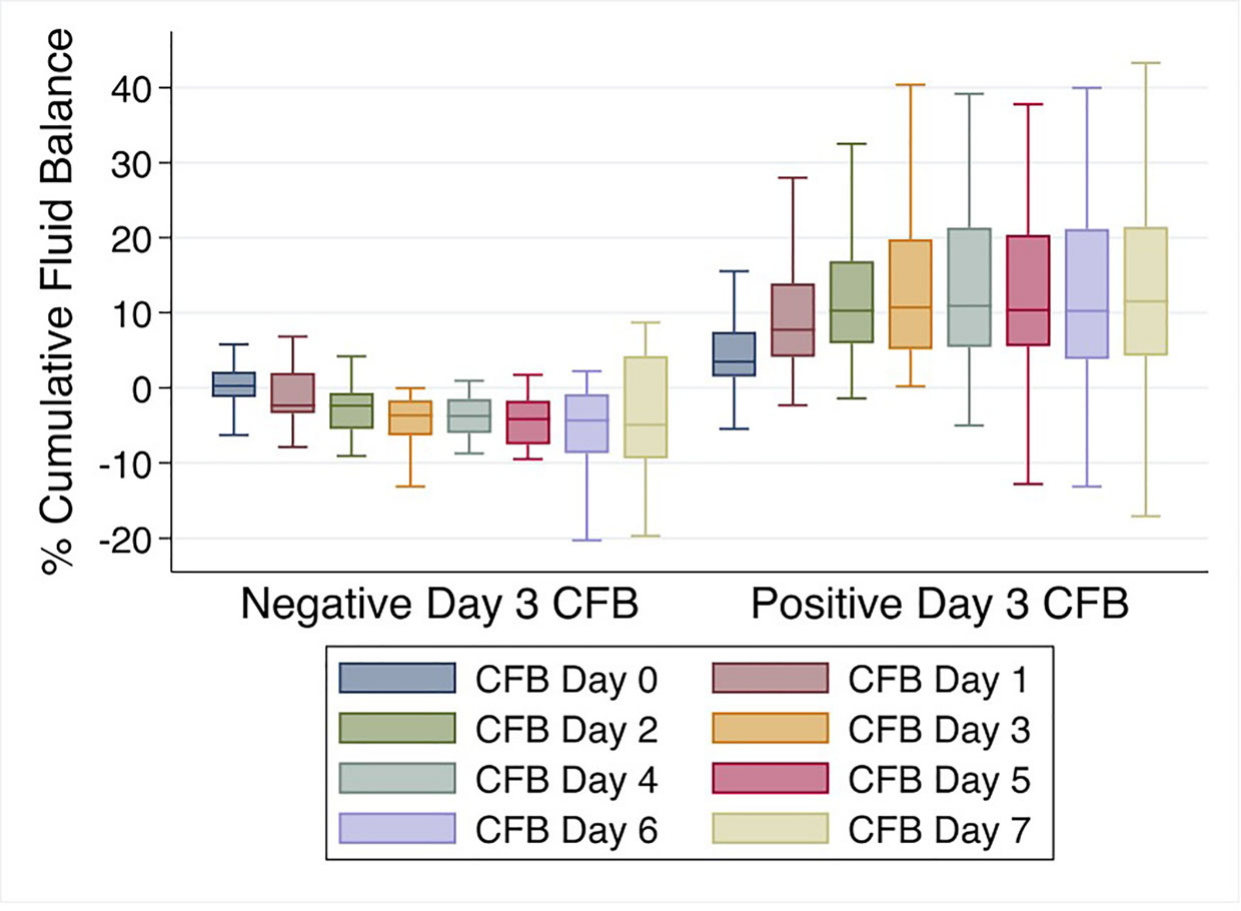
% Cumulative fluid balance over first week of invasive mechanical ventilation comparing negative *vs.* positive day 3 cumulative fluid balance groups.

Mean % CFB for the entire cohort was positive on day of intubation (day 0) and increased further on days 1-7 of IMV. Mean % CFB for IMV day 1, 2, 3, and 7 were +7.5% ( ± 8.4%), +8.8% ( ± 10.2%), +9.2% ( ± 11.9%), and +11.5.% ( ± 17.3%), respectively (**Figure 3**). Patients with negative day 3 CFB had a mean % CFB = -5.2% ( ± 7.8%), and those with positive day 3 CFB had mean % CFB = +12.9% ( ± 9.8%). Additionally, patients with negative day 3 CFB continued to have negative CFB throughout the first week of IMV. In contrast, among those with positive day 3 CFB, CFB remained positive (**Figure 4**).

### Primary Outcome: PICU Mortality

The overall PICU mortality in the cohort was 61% (n = 121/198). Mortality was 46% (n = 17/37) among patients with negative day 3 CFB and 65% (n = 104/161) among those with positive day 3 CFB. When stratified by CFB categories, mortality was 63% (48/76), 65% (31/48), and 68% (25/37) among patients with ≥0 to <10%, ≥10 to <20%, and ≥20% CFB, respectively.

The association between day 3 CFB and PICU mortality was analyzed with both bivariate and multivariable analyses (**Table 2**). Multivariable analysis showed there was a strong association between increasing % day 3 CFB and a higher odds of PICU mortality. When adjusting for the covariates, for each 1% increase in day 3 CFB, the odds of PICU mortality were higher by 3% (aOR 1.03, 95% CI 1.00-1.07, p<0.05).

**Table 2:** Association between day 3 cumulative fluid balance and PICU mortality.

Exposure: Day 3 IMV	Unadjusted OR	*p* ^a^	Adjusted OR^b^	*p* ^a^
	(95% CI)		(95% CI)	
% CFB	1.02 (1.00-1.05)	0.057	1.03 (1.00-1.07)	**<0.05**
Positive CFB	2.15 (1.04-4.42)	**<0.05**	3.42 (1.48-7.87)	**<0.01**
Categorical CFB^c^	1.30 (0.97-1.74)	0.079	1.49 (1.06-2.10)	**<0.05**
(test of trend)				
Categorical CFB				
Negative CFB	Reference		Reference	
≥0-<10%	2.02 (0.91-4.48)	0.085	3.11 (1.25-7.75)	**<0.05**
≥10-<20%	2.15 (0.89-5.15)	0.088	3.81 (1.40-10.36)	**<0.01**
≥20%	2.45 (0.95-6.30)	0.063	3.64 (1.22-10.87)	**<0.05**

OR, odds ratio represented with 95% confidence interval; CFB, cumulative fluid balance; IMV, invasive mechanical ventilation.

a bold = statistically significant at α level 0.05.

b Multivariable logistic regression models were adjusted for age, sex, days from hematopoietic cell transplantation to PICU admission, presence of positive respiratory pathogen, and receipt of renal replacement therapy, inhaled nitric oxide, and vasopressors/inotropes.

c CFB categories = negative, ≥0 to <10%, ≥10 to <20%, or ≥20%.

Furthermore, when day 3 CFB was examined dichotomously, positive day 3 CFB was independently associated with a 3.42 times higher odds of PICU mortality (95% CI 1.48-7.87, p<0.01). Relative to negative day 3 CFB, the odds of mortality were 2.15 times higher for ≥10-<20% and 2.45 times higher for ≥20% on bivariate analysis. On multivariable analysis and relative to the negative day 3 CFB group, the odds of mortality were 3.81 fold higher for ≥10-<20% and 3.64 fold higher for ≥20% (**Table 2**). When CFB categories were analyzed in an ordinal fashion, there was evidence of linear trend. The multivariable analysis showed that the odds of PICU mortality were higher by a factor of 1.49 for each increase in CFB category (95% CI 1.06-2.10, p<0.05). In other words, the odds of PICU mortality were almost 50% higher comparing adjacent CFB categories (i.e. negative to ≥0- <10%, ≥0-<10% to ≥10-<20%, and ≥10-<20% to ≥20% CFB).

### Secondary Outcomes: Rate of Extubation at 28 Days and 60 Days

After accounting for the competing event of PICU mortality, day 3 CFB, analyzed as a continuous variable, was associated with the rate of extubation at 28 and 60 days on bivariate and multivariable analyses (**Tables 3** and **4**). After adjusting for the covariates, % day 3 CFB was associated with a lower rate of extubation. For each 1% increase in day 3 CFB, the rate of extubation was lower by 3% at 28 days (aSHR 0.97, 95% CI 0.95-0.98, p<0.001) and 3% at 60 days (aSHR 0.97, 95% CI 0.95-0.98, p<0.001).

**Table 3:** Association between day 3 cumulative fluid balance and rate of extubation (28 days).

Exposure: Day 3 IMV	Unadjusted SHR	*p* ^a^	Adjusted SHR^b^	*p* ^a^
	(95% CI)		(95% CI)	
% CFB	0.97 (0.96-0.99)	**<0.001**	0.97 (0.95-0.98)	**<0.001**
Positive CFB	0.45 (0.27-0.74)	**<0.01**	0.30 (0.18-0.48)	**<0.001**
Categorical CFB^c^	0.72 (0.57-0.92)	**<0.01**	0.64 (0.49-0.82)	**<0.001**
(test of trend)				
Categorical CFB				
Negative CFB	Reference		Reference	
≥0-<10%	0.49 (0.28-0.88)	**<0.05**	0.32 (0.18-0.59)	**<0.001**
≥10-<20%	0.47 (0.25-0.86)	**<0.05**	0.28 (0.16-0.49)	**<0.001**
≥20%	0.36 (0.18-0.72)	**<0.01**	0.26 (0.13-0.52)	**<0.001**

SHR, subdistribution hazard ratio represented with 95% confidence interval; CFB, cumulative fluid balance; IMV, invasive mechanical ventilation.

a bold = statistically significant at α level 0.05.

b Multivariable logistic regression models were adjusted for age, sex, days from hematopoietic cell transplantation to PICU admission, presence of positive respiratory pathogen, and receipt of renal replacement therapy, inhaled nitric oxide, and vasopressors/inotropes. Data was censored at 28 days.

c CFB categories = negative, ≥0 to <10%, ≥10 to <20%, or ≥20%.

**Table 4:** Association between day 3 cumulative fluid balance and rate of extubation (60 days).

Exposure: Day 3 IMV	Unadjusted SHR	*p* ^a^	Adjusted SHR^b^	*p* ^a^
	(95% CI)		(95% CI)	
% CFB	0.97 (0.96-0.99)	**<0.01**	0.97 (0.95-0.98)	**<0.001**
Positive CFB	0.47 (0.29-0.76)	**<0.01**	0.30 (0.19-0.48)	**<0.001**
Categorical CFB^c^	0.73 (0.59-0.91)	<0.01	0.65 (0.51-0.83)	**<0.001**
(test of trend)				
Categorical CFB				
Negative CFB	Reference		Reference	
≥0-<10%	0.52 (0.30-0.90)	**<0.05**	0.34 (1.19-7.16)	**<0.001**
≥10-<20%	0.47 (0.26-0.84)	**<0.05**	0.28 (1.31-9.42)	**<0.001**
≥20%	0.37 (0.19-0.72)	**<0.01**	0.27 (1.06-8.59)	**<0.001**

SHR, subdistribution hazard ratio represented with 95% confidence interval; CFB, cumulative fluid balance; IMV, invasive mechanical ventilation.

a bold = statistically significant at α level 0.05.

b Multivariable logistic regression models were adjusted for age, sex, days from hematopoietic cell transplantation to PICU admission, presence of positive respiratory pathogen, and receipt of renal replacement therapy, inhaled nitric oxide, and vasopressors/inotropes. Data was censored at 60 days.

c CFB categories = negative, ≥0 to <10%, ≥10 to <20%, or ≥20%.

**Figure 5:**
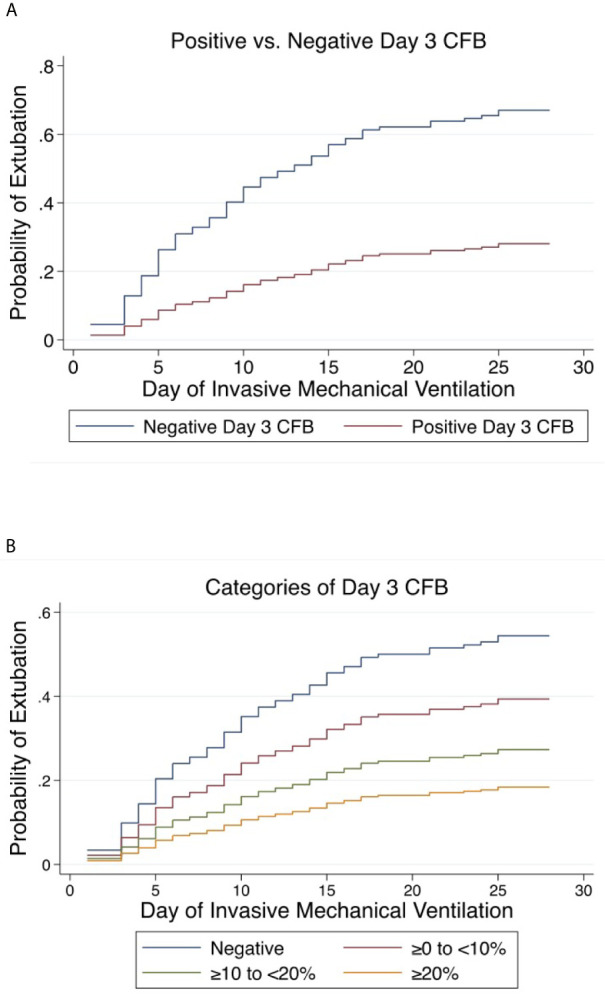
Cumulative incidence functions for extubation at 28 days. Competing risk = PICU mortality **(A)** Positive *vs.* Negative Day 3 Cumulative Fluid Balance. **(B)** Categories of Day 3 Cumulative Fluid Balance. CFB, Cumulative Fluid Balance.

Positive day 3 CFB was independently associated with a lower rate of extubation at 28 days (aSHR 0.30, 95% CI 0.183-0.478, p<0.001) and 60 days (aSHR 0.30, 95% 0.192-0.482, p<0.001) (**Figure 5A**). Linear trend was observed between CFB categories. For each increase in CFB category, the probability of extubation was significantly lower by 36% (aSHR 0.64, 95% CI 0.49-0.82, p<0.001) and 35% (aSHR 0.65, 95% CI 0.51-0.82, p<0.001) at 28 and 60 days on multivariable analysis (**Figure 5B**). Finally, relative to those with negative day 3 CFB, the rate of extubation was significantly lower in each other CFB category at 28 and 60 days (**Tables 3** and **4**).

### Treatment With RRT

75 patients (38%) received treatment with RRT. Overall PICU mortality was 72% (n = 54/75) compared to 54% (n = 67/123) among patients not treated with RRT. Patients with negative day 3 CFB received treatment with RRT more often (54% *vs.* 34%).

#### Impact of RRT on PICU Mortality

**Figure 6:**
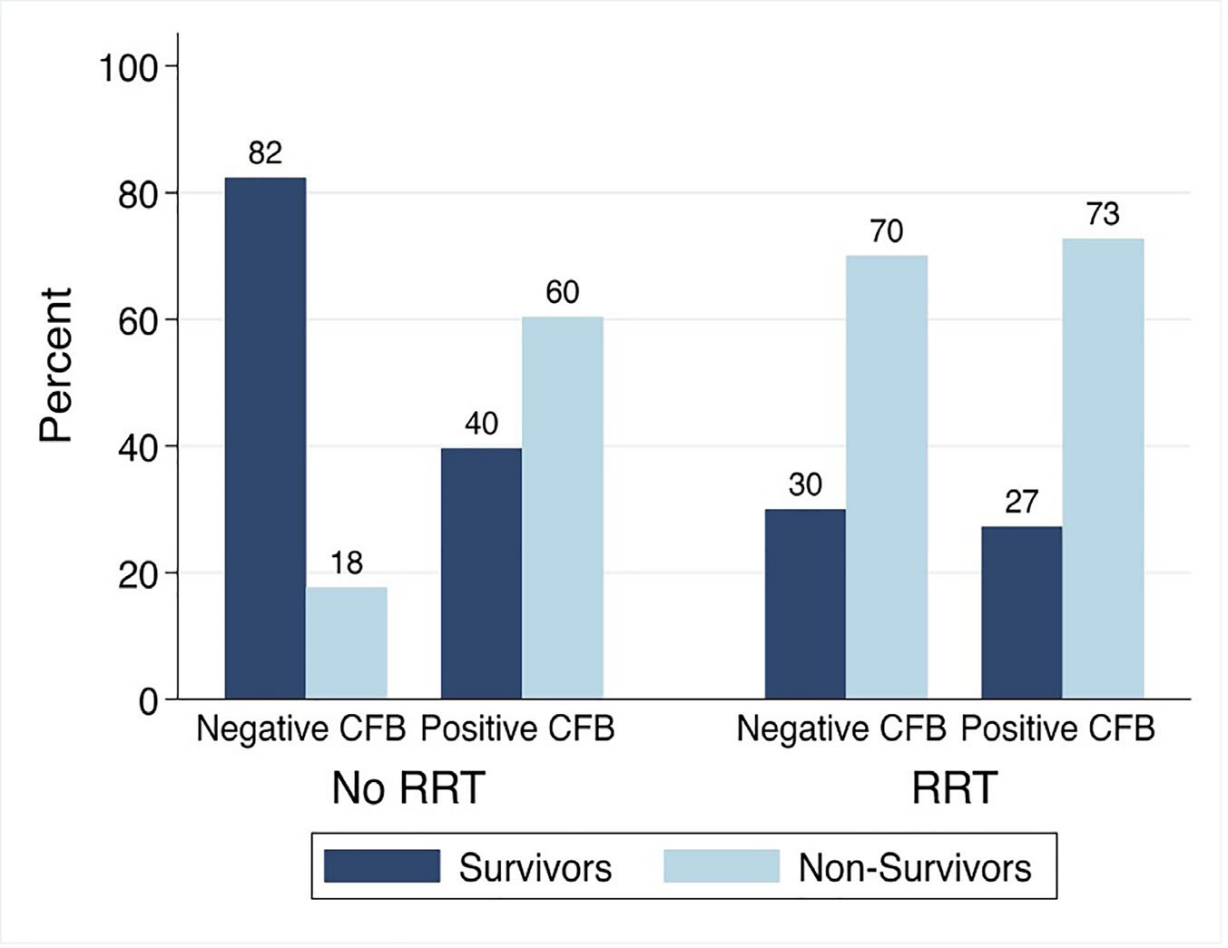
PICU Mortality by Day 3 Cumulative Fluid Balance Stratified by Treatment with Renal Replacement Therapy. CFB, cumulative fluid balance; RRT, renal replacement therapy; Non-Survivors, Patients with PICU Mortality.

Among patients not treated with RRT, the PICU mortality was 18% (3/17) in those with negative and 60% (64/106) in those with positive day 3 CFB. Among patients treated with RRT, the PICU mortality was 70% (14/20) and 73% (40/55) in those with negative and positive day 3 CFB (**Figure 6**).

The association between positive day 3 CFB and PICU mortality significantly varied by treatment with RRT (p<0.05). On multivariable analysis, the association between positive day 3 CFB and odds of PICU mortality was significantly greater among those not treated with RRT. Among patients not treated with RRT, the odds of PICU mortality were 9.11 times higher (95% CI 2.29-36.22) compared to 1.40 times higher (95% CI 0.42-4.74) in those treated with RRT.

#### Impact of RRT on Rate of Extubation at 28 Days and 60 Days

On multivariable analysis, there was not a significant interaction between treatment with RRT and day 3 CFB with respect to the rate of extubation at 28 days (p = 0.28) and 60 days (p = 0.08). Among those not treated with RRT and positive day 3 CFB the rate of extubation was 75% lower (aSHR 0.25, 95% CI 0.14-0.43) and 78% lower (aSHR 0.22, 95% CI 0.12-0.39) at 28 and 60 days, respectively. In contrast, the rate of extubation was 56% lower (aSHR 0.44, 95% CI 0.18-1.04) and 50% lower (aSHR 0.50, 95% CI 0.24-1.04) at 28 and 60 days among those treated with RRT and positive day 3 CFB. Although the magnitude of the associations between day 3 CFB and adverse outcomes were greater among those not treated with RRT, they were not statistically different from the associations seen among patients treated with RRT.

### Sensitivity Analysis: Excluding IMV Courses <3 Days

Few patients with IMV <3 days had negative day 3 CFB (2/26) and PICU mortality was high in this group at 81% (21/26). We performed a *post-hoc* sensitivity analysis excluding the 26 patients with courses of IMV <3 days. In the multivariable model, positive day 3 CFB remained associated with higher PICU mortality (aOR 2.70, 95% CI 1.12-6.49, p<0.05) and a lower rate of extubation at 28 days (aSHR 0.33, 95% CI 0.20-0.53, p<0.001), and 60 days (aSHR 0.35, 95% CI 0.22-0.56, p<0.001).

## Discussion

This analysis adds to the accumulating literature noting the contribution of early positive cumulative fluid balance to adverse outcomes in critically ill patients with acute respiratory failure. We were able to confirm these associations in 198 pediatric allogeneic HCT recipients with ARF requiring IMV in several complementary analyses. We found that early positive and increasing CFB were independently associated with higher PICU mortality and lower rate of extubation at 28 and 60 days. Not only was positive CFB an important threshold, there was evidence of a dose-response relationship between CFB and worse outcomes at our clinical cut points of negative, ≥0 to <10%, ≥10 to <20%, or ≥20% CFB. Finally, there was evidence that the relationship between early positive CFB and PICU mortality differed by receipt of RRT. We found that the consequence of early positive CFB with respect to PICU mortality was significantly more harmful among those not treated with RRT.

Our results demonstrate similar fluid balance metrics to both the Valentine et al. study (16) and liberal arm of the FACTT trial (20) in the first week of the respective study periods. By day 3 of the study period, mean CFB was +8.5% ( ± 10.5%) in the Valentine et al. study compared to +9.2% ( ± 11.9%) in our cohort. Measuring CFB early in the course of IMV has precedent in prior studies evaluating the association between CFB and outcomes in patients with ARF (12–14, 27, 28). Nevertheless, the threshold at which CFB becomes particularly harmful remains in question. Observational data in critically ill pediatric patients support prevention of at least >10-20% CFB (29–31). Previous work by Michael et al. (32) demonstrated that among pediatric HCT patients with acute renal failure, all survivors during their hospital course maintained <10% fluid balance or reattained <10% fluid balance with RRT treatment. Our results suggest that any positive day 3 fluid accumulation could be highly detrimental, and that in contrast, achieving a negative fluid balance early in the course of IMV could be of benefit. This is supported by the fact the negative day 3 CFB cohort remained negative in subsequent days during the first week of IMV. We also observed a dose-response relationship between increasing categories of day 3 CFB and more adverse outcomes. Our results emphasize the clinical relevance of the specified cut points of negative, ≥0 to <10%, ≥10 to <20%, and ≥20% CFB.

No pediatric or adult studies to date have demonstrated a causal contribution of fluid accumulation to mortality with some arguing that positive CFB may represent a prognostic event and mortality more reflective of underlying illness severity or shock. However, a meta-analysis by Alobaidi et al. (11) that included 11 studies of critically ill children (n = 3200), demonstrated that after adjustment for illness severity, there was a 6% increase in odds of mortality for every 1% increase in fluid balance (aOR 1.06, 95% CI 1.03-1.10). In the present investigation, after adjustment for the covariates, for each 1% increase in day 3 CFB, PICU mortality was 3% higher. Therefore, if it is well established that early positive CFB represents at least a harbinger of poor outcomes, the hope is that it can be a modifiable factor to prevent or at minimum, mitigate.

Although the relationship between positive fluid balance and mortality may be more nebulous, the relationship with lung disease is more compelling. The FACTT trial demonstrated that conservative fluid management improved lung function and increased ventilator free days (20). When Yehya et al. (33) applied competing risk regression to the FACTT trial, the rate of extubation was 30% higher among those in the conservative fluid management arm (SHR 1.30, 95% CI 1.12-1.51). As our exposure groups were hypothesized to have SHR <1, we were able to reproduce similar findings such that positive and increasing CFB were independently associated with a lower rate of extubation. The finding that our results persisted to 60 days underscores not only the importance of a CFB threshold, but the evolving emphasis of the timing of fluid accumulation and subsequent downstream respiratory outcomes (34).

The pediatric HCT recipient may be uniquely at risk for positive fluid accumulation during their PICU course. HCT recipients can require hyperhydration for chemotherapy, total parental nutrition, intravenous infusions for antibiotics, and frequent blood product transfusions. Furthermore, activation and subsequent dysfunction of the vascular endothelium has been implicated in triggering a number of life-threatening HCT-related complications (35–37). It has been proposed that complications such as transplant-associated thrombotic microangiopathy (TA-TMA), idiopathic pneumonia syndrome/diffuse alveolar hemorrhage (IPS/DAH), veno-occlusive disease/sinusoidal obstructive syndrome (VOD/SOS), and acute graft *versus* host disease (GVHD) can be traced back to endothelial cell activation (38). This activation can occur as a result of the chemo- and radio-therapy needed for conditioning (38, 39). The transition from endothelial activation to the phenotype of endothelial damage could be secondary to a dysregulated host immune response and inflammation that alters capillary permeability (40, 41). Presumptively, in the context of capillary leak, this may make conventional mechanisms of fluid management with diuretics and/or restrictive fluid strategies more challenging. Furthermore, the high incidence of acute kidney injury in this population only compounds the obstacles faced when approaching fluid management in the PICU setting (42).

RRT has been suggested to have a role in mitigating the adverse impacts of positive CFB on outcomes in critically ill pediatric HCT recipients. Recently, Raymakers-Janssen et al. (23) demonstrated that among 68 pediatric patients with cancer and post-HCT, the odds of mortality were 6.2 times higher among those with >10% CFB at RRT initiation. However, data regarding the role of RRT in those with ARF is scarce. Elbahlawan et al. (43) demonstrated among pediatric HCT recipients with ARF, oxygenation and concurrently fluid balance improved with initiation of RRT. However, it was unclear if the oxygenation benefits were sustained or conferred any meaningful survival benefit. In a case series by DiCarlo et al. (44) among 10 immunocompromised children (of whom 6 were post-HCT) with ARF receiving early RRT, 9 were successfully extubated and 8 survived. We observed that the negative day 3 CFB group was more likely to be treated with RRT. However, mortality estimates remained around 70% independent of positive or negative day 3 CFB in those treated with RRT. High mortality in the RRT cohort may be more reflective of high illness severity, presence of multiple organ dysfunction, uncontrolled infection, or disease relapse. Given that we do not know the timing, indication, duration, specific modality of RRT or concurrent kidney dysfunction, we cannot draw strong conclusions regarding treatment with RRT and subsequent patient outcomes. On the other hand, positive day 3 CFB was significantly more harmful among those not treated with RRT. There was only 18% mortality among patients with negative day 3 CFB and no treatment with RRT. This may be an important group for which fluid balance was a truly modifiable risk factor. We can only speculate that these patients were either judiciously or actively managed with respect to fluid balance and perhaps lacked the concurrent organ dysfunction so characteristic of this patient population (45). The association between day 3 CFB and rate of extubation did not significantly vary by treatment with RRT. Nonetheless, the magnitude of the association between positive day 3 CFB and lower rates of extubation were greater in the non-RRT group.

The study has notable limitations. We cannot account for fluid management prior to admission to the PICU or prior to intubation. While study day 0 was day of intubation in the current study, in the original investigation of this database, median length of PICU stay prior to intubation was 0 days (IQR 0-2) with 58% being intubated on the same day of PICU admission (5). Therefore, fluid management in the time between PICU admission and intubation may have influenced outcomes, but this period was quite short in over half of the cohort. Ultimately, the impact of fluid management on outcomes in this population prior to admission to the PICU is an ongoing research consideration. As this was a retrospective secondary analysis, there is risk for residual confounding. Illness severity and organ dysfunction scores were not present in original dataset. We addressed this limitation by adjusting for variables ostensibly reflective of illness severity, such as treatment with vasoactive or inotropic infusions, iNO, and RRT as well as presence of a positive respiratory pathogen. Finally, our study population was sampled between 2009 and 2014. Therefore, practices regarding fluid management in our cohort may be more reflective of historical precedent rather than current practice.

The present investigation has many strengths. We conducted a multicenter study with a database that provided granular data on a patient population that frequently experiences critical illness. We were able to demonstrate similar directions of association with multiple complementary analyses, which persisted even with removal of the 26 patients with IMV courses <3 days. We were able to apply a novel approach to analyzing duration of IMV using competing risk regression and isolating the association between CFB and extubation, accounting for the competing risk of death. Given the large sample size and multicenter nature of the investigation, these results could be generalizable to pediatric allogeneic HCT recipients with ARF requiring intensive care support.

In this multicenter cohort, early positive and increasing CFB were independently associated with higher PICU mortality and a lower rate of extubation among pediatric allogeneic HCT recipients with ARF. The association between early positive CFB with PICU mortality was significantly stronger in those not receiving RRT. These results suggest that conservative fluid management early in the course of IMV may improve outcomes. The role of RRT in managing fluid balance in this vulnerable population with ARF requires further investigation.

## Data Availability Statement

This is not a publicly available dataset. The data will be available upon request. Requests to access these datasets should be directed to coujohns@iu.edu.

## Ethics Statement

Ethical review and approval were not required for the study on human participants in accordance with the local legislation and institutional requirements. Written informed consent from the participants’ legal guardian/next of kin was not required to participate in this study in accordance with the national legislation and the institutional requirements.

## Author Contributions

CS contributed to the conception and design of the work, the analysis and interpretation of the work, and drafted the work. LS and SH contributed to the conception and design of the work, the analysis and interpretation of the work, and revising the work critically for important intellectual content. JF and CR contributed to the conception and design of the work, data acquisition, the analysis and interpretation of the work, and revising the work critically for important intellectual content. JA contributed to the design of the work, the interpretation of the work, and revising the work critically for important intellectual content. MD, SG, DH, KM, and JM contributed to the conception of the work, data acquisition, the interpretation of the work, and revising the work critically for important intellectual content. All authors provided final approval of the version to be published and agree to be accountable for all aspects of the work in ensuring that questions related to the accuracy or integrity of any part of the work are appropriately investigated and resolved. All authors contributed to the article and approved the submitted version.

## Funding

JF is supported by NIH NIDDK K23 DK119463. CR is supported by NHLBI K23 HL150244.

## Conflict of Interest

The authors declare that the research was conducted in the absence of any commercial or financial relationships that could be construed as a potential conflict of interest.
